# Supplementary material for: Bartonella effector protein C mediates actin stress fiber formation via recruitment of GEF-H1 to the plasma membrane
Source: PLoS Pathog. 2021 Jan 28;17(1):e1008548. doi: 10.1371/journal.ppat.1008548 (PMC7842960; doi:10.1371/journal.ppat.1008548)
Supplement: S1 Fig — (A, B) HUVECs were infected with isogenic Bhe ΔbepA-G strains expressing 3xFLAG-tagged BepCBhe wild-type or mutant versions or carrying the empty plasmid at indicated MOIs for 24 or 48 h. After fixation, cells were stained by immunocytochemistry, followed by fluorescence microscopy analysis. (A) Shown are representative images for Bhe ΔbepA-G strains expressing 3xFLAG-tagged BepCBhe wild-type and the isogenic empty plasmid control. F-actin is represented in green, DNA in blue, and bacteria in red (scale bar = 50 μm). (B) The graphs show the relative mean fluorescence intensity of the F-actin signal at 24 hpi (left panel) and 48h (right panel) for the indicated MOIs normalized to the uninfected control. Shown are results from three independent experiments. BepCBhe**** = BepCBhe H146A, K150A, R154A, R157A; BepCBhe (Flap BepABhe) = BepCBhe A90E, R92K, P93R, K94T, H96W, R97K, V98N, P99A; BepCBhe (OB-BID) = BepCBhe Δ1–226. (PDF) [file ppat.1008548.s001.pdf]

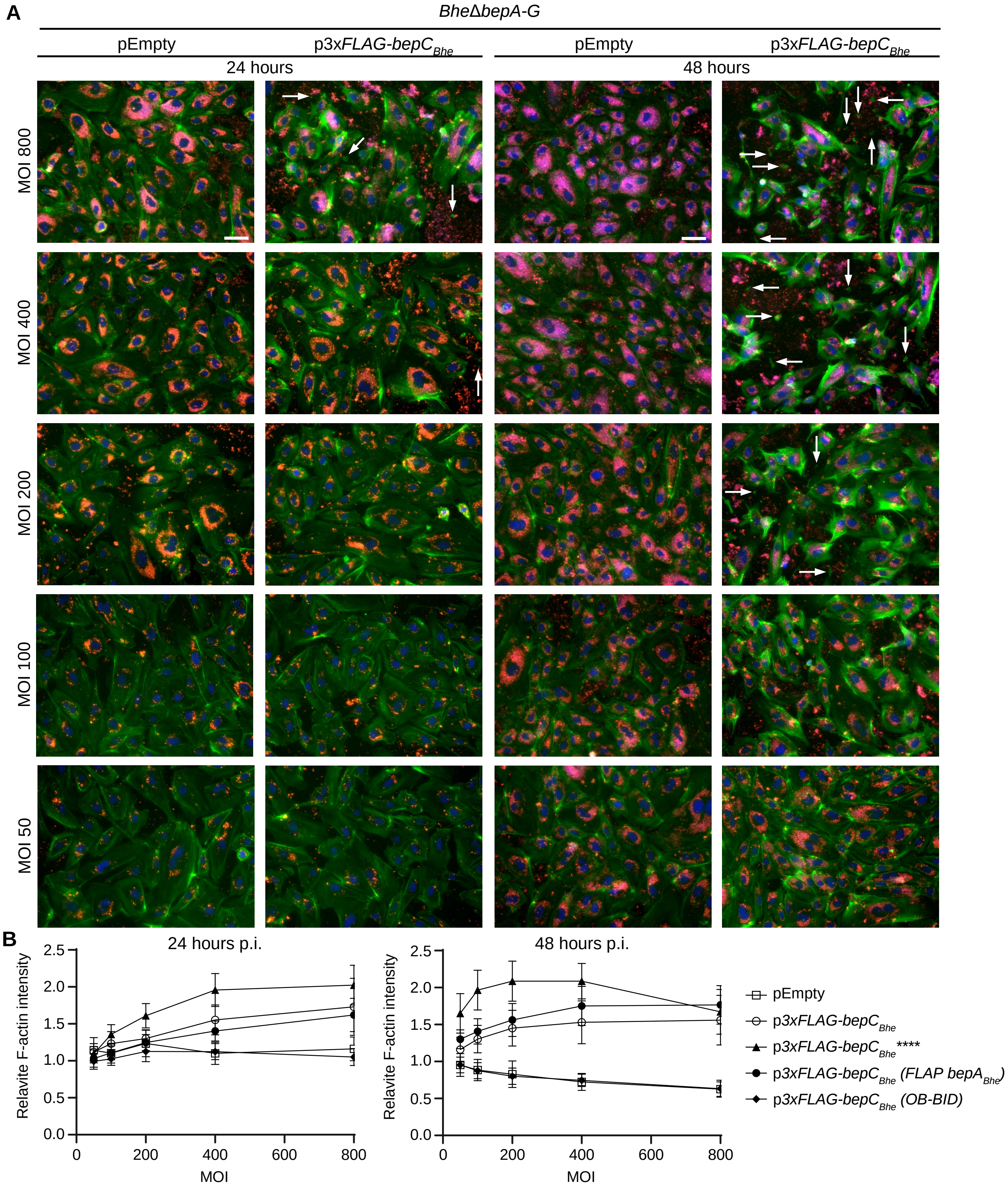
A
BheΔbepA-G
pEmpty
p3xFLAG-bepC_Bhe
pEmpty
p3xFLAG-bepC_Bhe
24 hours
48 hours
MOI 800
MOI 400
MOI 200
MOI 100
MOI 50
B
24 hours p.i.
48 hours p.i.
Relavite F-actin intensity
MOI
0.0
0.5
1.0
1.5
2.0
2.5
0
200
400
600
800
pEmpty
p3xFLAG-bepC_Bhe
p3xFLAG-bepC_Bhe ****
p3xFLAG-bepC_Bhe (FLAP bepA_Bhe)
p3xFLAG-bepC_Bhe (OB-BID)

S1 Fig. BepC$_{Bhe}$-triggered actin stress fiber formation in infected *B. henselae*-infected HUVEC is dependent on time and multiplicity of infection. (**A, B**) HUVECs were infected with isogenic *Bhe* Δ*bepA-G* strains expressing 3xFLAG-tagged BepC$_{Bhe}$ wild-type or mutant versions or carrying the empty plasmid at indicated MOIs for 24 or 48 h. After fixation, cells were stained by immunocytochemistry, followed by fluorescence microscopy analysis. (**A**) Shown are representative images for *Bhe* Δ*bepA-G* strains expressing 3xFLAG-tagged BepC$_{Bhe}$ wild-type and the isogenic empty plasmid control. F-actin is represented in green, DNA in blue, and bacteria in red (scale bar = 50 µm). (**B**) The graphs show the relative mean fluorescence intensity of the F-actin signal at 24 hpi (left panel) and 48h (right panel) for the indicated MOIs normalized to the uninfected control. Shown are results from three independent experiments. BepC$_{Bhe}$**** = BepC$_{Bhe}$ H146A, K150A, R154A, R157A; BepC$_{Bhe}$ (Flap bepA) = BepC$_{Bhe}$ A90E, R92K, P93R, K94T, H96W, R97K, V98N, P99A; BepC$_{Bhe}$ (OB-BID) = BepC$_{Bhe}$ Δ1-226.
