# Supplementary material for: Bartonella effector protein C mediates actin stress fiber formation via recruitment of GEF-H1 to the plasma membrane
Source: PLoS Pathog. 2021 Jan 28;17(1):e1008548. doi: 10.1371/journal.ppat.1008548 (PMC7842960; doi:10.1371/journal.ppat.1008548)
Supplement: S2 Fig — (A) HeLa cells were infected with isogenic Bhe ΔbepA-G strains expressing FLAG-tagged BepCBhe wild-type or mutant versions or carrying the empty plasmid at multiplicity of infection (MOI) of 400. After 48 h of infection, cells were fixed and immunocytochemically stained with anti-FLAG antibody, followed by fluorescence microscopy analysis. FLAG staining is shown in white and corresponds to the images displayed in Fig 2A (scale bar = 50 μm). (B) HeLa cells were transfected with indicated plasmids for expression of FLAG-tagged BepCBhe wild-type, mutant versions, or truncations, or no protein as negative control (pEmpty). 24 h after transfection, cells were fixed and immunocytochemically stained, followed by fluorescence microscopic analysis. FLAG staining is represented in white and corresponds to the images displayed in Fig 3B (scale bar = 50 μm). BepCBhe**** = BepCBhe H146A, K150A, R154A, R157A. Shown are representative results of three independent experiments. (PDF) [file ppat.1008548.s002.pdf]

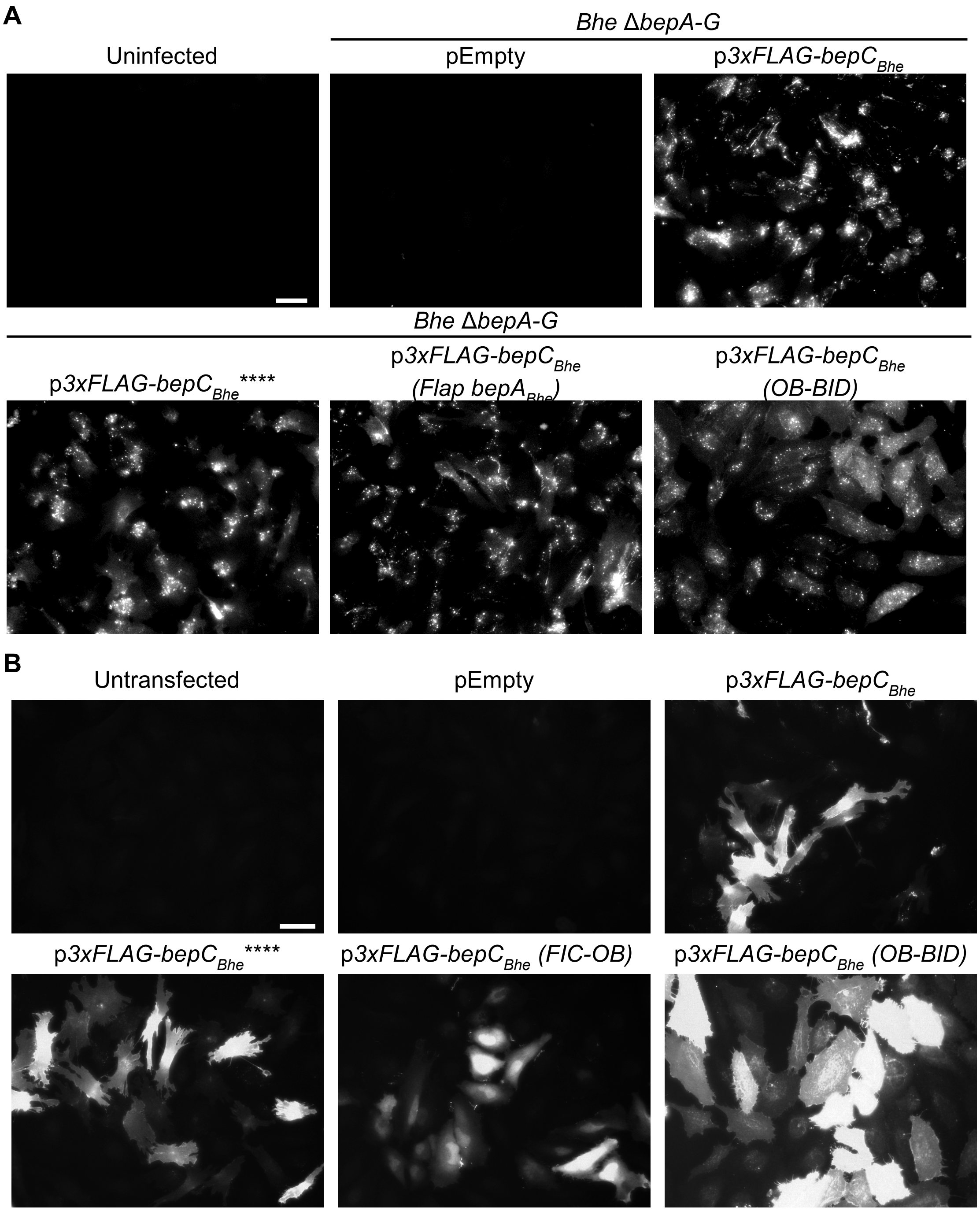
A
Bhe ΔbepA-G
Uninfected
pEmpty
p3xFLAG-bepC_Bhe
Bhe ΔbepA-G
p3xFLAG-bepC_Bhe****
p3xFLAG-bepC_Bhe (Flap bepA_Bhe)
p3xFLAG-bepC_Bhe (OB-BID)
B
Untransfected
pEmpty
p3xFLAG-bepC_Bhe
p3xFLAG-bepC_Bhe****
p3xFLAG-bepC_Bhe (FIC-OB)
p3xFLAG-bepC_Bhe (OB-BID)

S2 Fig. Expression of 3xFLAG-tagged BepC*Bhe* in infected and transfected HeLa cells. (**A**) HeLa cells were infected with isogenic *Bhe* Δ*bepA-G* strains expressing FLAG-tagged BepC*Bhe* wild-type or mutant versions or carrying the empty plasmid at multiplicity of infection (MOI) of 400. After 48 h of infection, cells were fixed and immunocytochemically stained with anti-FLAG antibody, followed by fluorescence microscopy analysis. FLAG staining is shown in white and corresponds to the images displayed in Fig 2A (scale bar = 50 µm). (**B**) HeLa cells were transfected with indicated plasmids for expression of FLAG-tagged BepC*Bhe* wild-type, mutant versions, or truncations, or no protein as negative control (pEmpty). 24 h after transfection, cells were fixed and immunocytochemically stained, followed by fluorescence microscopic analysis. FLAG staining is represented in white and corresponds to the images displayed in Fig 3B (scale bar = 50 µm). BepC*Bhe***** = BepC*Bhe* H146A, K150A, R154A, R157A. Shown are representative results of three independent experiments.
