# Supplementary material for: Bartonella effector protein C mediates actin stress fiber formation via recruitment of GEF-H1 to the plasma membrane
Source: PLoS Pathog. 2021 Jan 28;17(1):e1008548. doi: 10.1371/journal.ppat.1008548 (PMC7842960; doi:10.1371/journal.ppat.1008548)
Supplement: S3 Fig — (A) HeLa were infected with Bhe ΔbepA-G or Bhe ΔbepA-G, ΔvirB4 expressing 3xFLAG-tagged BepCBhe or carrying empty plasmid as a negative control at MOI 400 for 48 h. After fixation, cells were stained by immunocytochemistry, followed by fluorescence microscopy analysis. F-actin is represented in green, DNA in blue, and bacteria in red (scale bar = 50 μm). (B) Expression of 3xFLAG-tagged BepCBhe in Bhe ΔbepA-G and Bhe ΔbepA-G, ΔvirB4 was analyzed by immunoblot using an anti-FLAG antibody. (C) The mean fluorescence intensity of F-actin shown for conditions shown in (A) were quantified for each individual cell using CellProfiler. Data are represented as dot plots with each data point corresponding to the average of all mean cell intensity values within one imaged site normalized to the uninfected control. Statistical significance was determined using Kruskal-Wallis test (**** corresponds to p-value ≤ 0.0001). (D) Corresponding FLAG channel of conditions shown in (A). FLAG staining is represented in white (scale bar = 50 μm). Data show a representative example of three independent experiments. (PDF) [file ppat.1008548.s003.pdf]

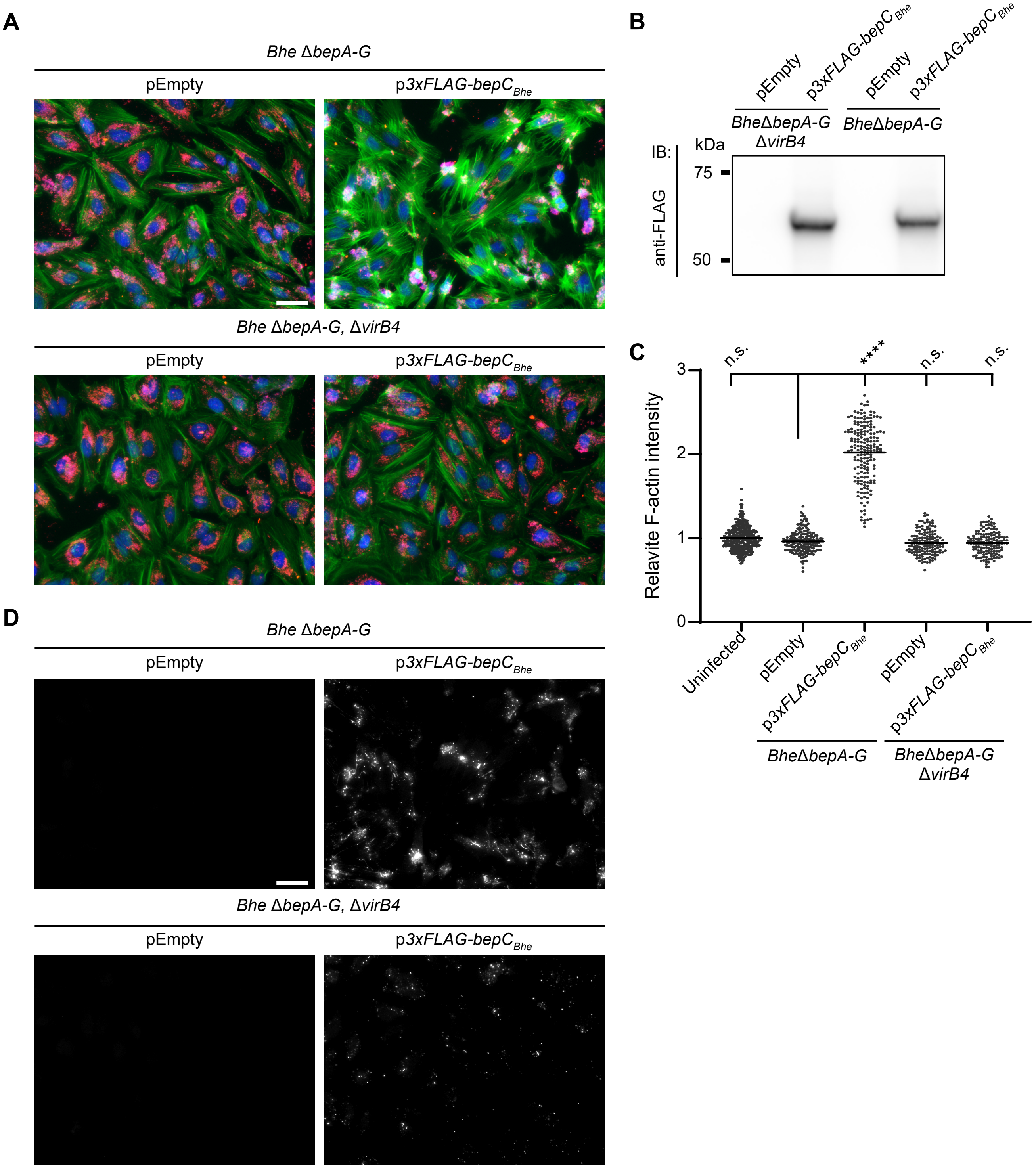
A
Bhe ΔbepA-G
pEmpty
p3xFLAG-bepC Bhe
Bhe ΔbepA-G, ΔvirB4
pEmpty
p3xFLAG-bepC Bhe
B
pEmpty
p3xFLAG-bepC Bhe
pEmpty
p3xFLAG-bepC Bhe
BheΔbepA-G ΔvirB4
BheΔbepA-G
IB:
kDa
75
50
anti-FLAG
C
n.s.
****
n.s.
n.s.
Relavite F-actin intensity
3
2
1
0
Uninfected
pEmpty
p3xFLAG-bepC Bhe
pEmpty
p3xFLAG-bepC Bhe
BheΔbepA-G
BheΔbepA-G ΔvirB4
D
Bhe ΔbepA-G
pEmpty
p3xFLAG-bepC Bhe
Bhe ΔbepA-G, ΔvirB4
pEmpty
p3xFLAG-bepC Bhe

S3 Fig. The BepC$_{Bhe}$-triggered actin stress fiber formation phenotype in *B. henselae*-infected HeLa cells is type-IV-secretion-dependent. (**A**) HeLa were infected with *Bhe* Δ*bepA-G or Bhe* Δ*bepA-G,* Δ*virB4* expressing 3xFLAG-tagged BepC$_{Bhe}$ or carrying empty plasmid as a negative control at MOI 400 for 48 h. After fixation, cells were stained by immunocytochemistry, followed by fluorescence microscopy analysis. F-actin is represented in green, DNA in blue, and bacteria in red (scale bar = 50 µm). (**B**) Expression of 3xFLAG-tagged BepC$_{Bhe}$ in *Bhe* Δ*bepA-G* and *Bhe* Δ*bepA-G,* Δ*virB4* was analyzed by immunoblot using an anti-FLAG antibody. (**C**) The mean fluorescence intensity of F-actin shown for conditions shown in (A) were quantified for each individual cell using CellProfiler. Data are represented as dot plots with each data point corresponding to the average of all mean cell intensity values within one imaged site normalized to the uninfected control. Statistical significance was determined using Kruskal-Wallis test (**** corresponds to p-value ≤ 0.0001). (**D**) Corresponding FLAG channel of conditions shown in (A). FLAG staining is represented in white (scale bar = 50 µm). Data show a representative example of three independent experiments**.**
