# Supplementary material for: Bartonella effector protein C mediates actin stress fiber formation via recruitment of GEF-H1 to the plasma membrane
Source: PLoS Pathog. 2021 Jan 28;17(1):e1008548. doi: 10.1371/journal.ppat.1008548 (PMC7842960; doi:10.1371/journal.ppat.1008548)
Supplement: S4 Fig — (A) HeLa cells were infected with the indicated isogenic Bhe ΔbepA-G strains expressing FLAG-tagged BepC homologs at MOI of 400. After 48 h cells were fixed and immunocytochemically stained, followed by fluorescence microscopy analysis. F-actin is represented in green, DNA in blue, and bacteria in red (scale bar = 50 μm). (B) Expression of FLAG-tagged BepC homologues in Bhe ΔbepA-G was analysed in bacterial lysates by immunoblot analysis with an anti-FLAG antibody. (C) The mean fluorescence intensity of F-actin shown for conditions shown in (A) was quantified for each individual cell using CellProfiler. Data are represented as dot plots with each data point corresponding to the average of all mean cell intensity values within one imaged site. Statistical significance was determined using Kruskal-Wallis test (**** corresponds to p-value ≤ 0.0001). (D) HeLa cells were transfected for 24h with indicated expression plasmids encoding different BepC homologs. Cells were fixed and immunocytochemically stained, followed by fluorescence microscopy analysis. F-actin is represented in green and DNA in blue (scale bar = 50 μm). (E) Expression of FLAG-tagged BepC homologues was analysed in cellular lysates by immunoblot with an anti-FLAG antibody. (F) The mean fluorescence intensity of F-actin shown for conditions shown in (D) was quantified for each individual cell using CellProfiler. Data are represented as dot plots with each data point corresponding to the average of all mean cell intensity values within one imaged site. Statistical significance was determined using Kruskal-Wallis test (**** corresponds to p-value ≤ 0.0001). Data show a representative example of three independent experiments. Bhe (B. henselae); Bqu (B. quintana); Btr (B. tribocorum); Bta (B. taylorii); Bgr (B. grahamii). (PDF) [file ppat.1008548.s004.pdf]

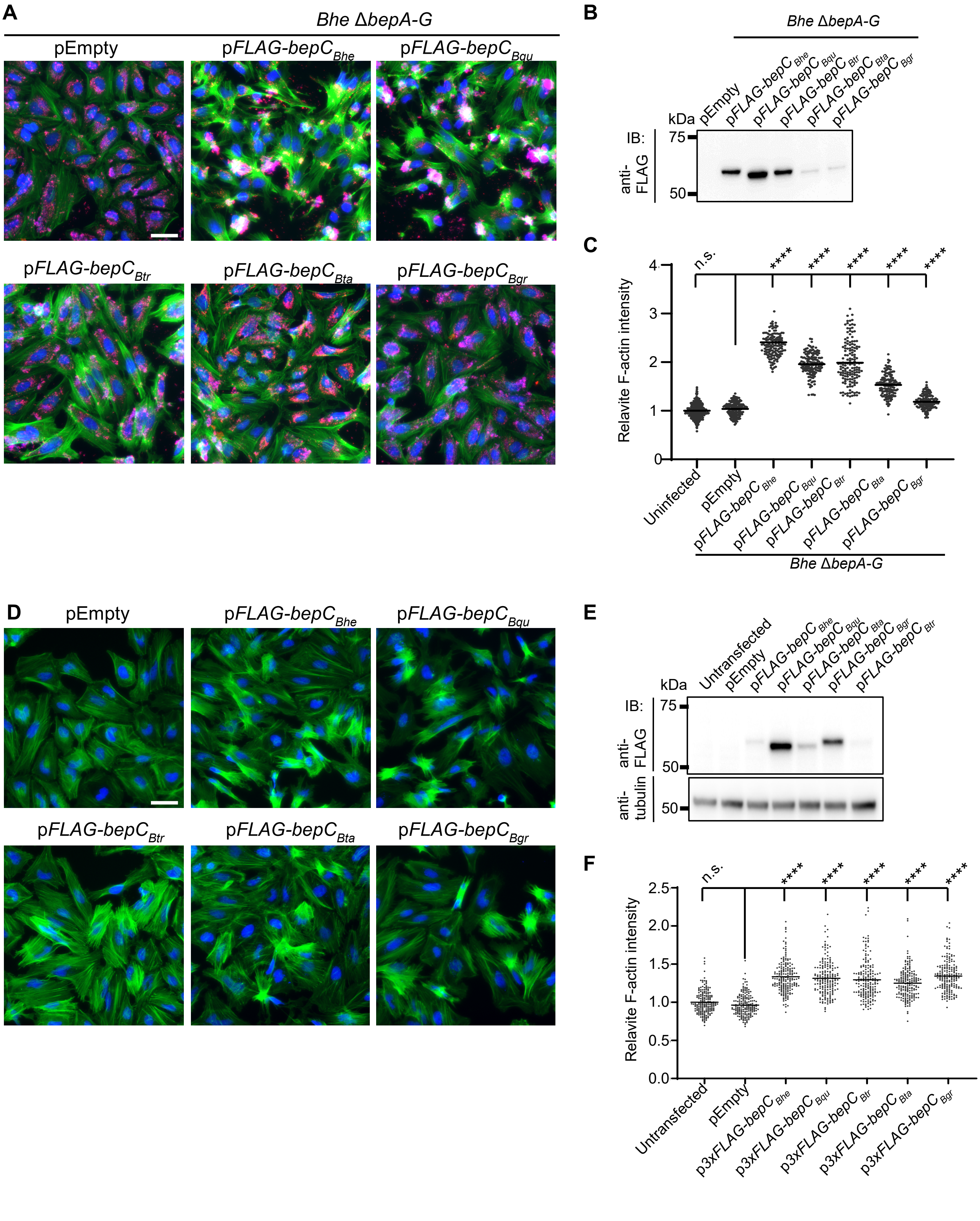
A
Bhe ΔbepA-G
pEmpty
pFLAG-bepCBhe
pFLAG-bepCBqu
pFLAG-bepCBtr
pFLAG-bepCBta
pFLAG-bepCBgr
B
Bhe ΔbepA-G
kDa
pEmpty
pFLAG-bepC Bhe
pFLAG-bepC Bqu
pFLAG-bepC Btr
pFLAG-bepC Bta
pFLAG-bepC Bgr
IB:
anti-FLAG
75
50
C
n.s.
****
Relavite F-actin intensity
0
1
2
3
4
Uninfected
pEmpty
pFLAG-bepC Bhe
pFLAG-bepC Bqu
pFLAG-bepC Btr
pFLAG-bepC Bta
pFLAG-bepC Bgr
Bhe ΔbepA-G
D
pEmpty
pFLAG-bepCBhe
pFLAG-bepCBqu
pFLAG-bepCBtr
pFLAG-bepCBta
pFLAG-bepCBgr
E
kDa
Untransfected
pEmpty
pFLAG-bepC Bhe
pFLAG-bepC Bqu
pFLAG-bepC Bta
pFLAG-bepC Bgr
pFLAG-bepC Btr
IB:
anti-FLAG
anti-tubulin
75
50
F
n.s.
****
Relavite F-actin intensity
0.0
0.5
1.0
1.5
2.0
2.5
Untransfected
pEmpty
p3xFLAG-bepC Bhe
p3xFLAG-bepC Bqu
p3xFLAG-bepC Btr
p3xFLAG-bepC Bta
p3xFLAG-bepC Bgr

S4 Fig. BepC-triggered actin stress fiber formation is conserved among homologs encoded by various *Bartonella* species. (A) HeLa cells were infected with the indicated isogenic *Bhe* Δ*bepA-G* strains expressing FLAG-tagged BepC homologs at MOI of 400. After 48 h cells were fixed and immunocytochemically stained, followed by fluorescence microscopy analysis. F-actin is represented in green, DNA in blue, and bacteria in red (scale bar = 50 μm). (**B**) Expression of FLAG-tagged BepC homologues in *Bhe* Δ*bepA-G* was analysed in bacterial lysates by immunoblot analysis with an anti-FLAG antibody. (**C**) The mean fluorescence intensity of F-actin shown for conditions shown in (A) was quantified for each individual cell using CellProfiler. Data are represented as dot plots with each data point corresponding to the average of all mean cell intensity values within one imaged site. Statistical significance was determined using Kruskal-Wallis test (**** corresponds to p-value ≤ 0.0001). (**D**) HeLa cells were transfected for 24h with indicated expression plasmids encoding different BepC homologs. Cells were fixed and immunocytochemically stained, followed by fluorescence microscopy analysis. F-actin is represented in green, DNA in blue, and bacteria in red (scale bar = 50 μm). (**E**) Expression of FLAG-tagged BepC homologues was analysed in cellular lysates by immunoblot with an anti-FLAG antibody. (**F**) The mean fluorescence intensity of F-actin shown for conditions shown in (D) was quantified for each individual cell using CellProfiler. Data are represented as dot plots with each data point corresponding to the average of all mean cell intensity values within one imaged site. Statistical significance was determined using Kruskal-Wallis test (**** corresponds to p-value ≤ 0.0001). Data show a representative example of three independent experiments**.** *Bhe* (*B. henselae*); *Bqu* (*B. quintana*); *Btr* (*B. tribocorum*); *Bta* (*B. taylorii*); *Bgr* (*B. grahamii*).
