# Supplementary material for: Bartonella effector protein C mediates actin stress fiber formation via recruitment of GEF-H1 to the plasma membrane
Source: PLoS Pathog. 2021 Jan 28;17(1):e1008548. doi: 10.1371/journal.ppat.1008548 (PMC7842960; doi:10.1371/journal.ppat.1008548)
Supplement: S5 Fig — (A) Proposed model of BepC-triggered actin stress fiber formation via the activation of the RhoA pathway and the targets of inhibitors used for validation. (B-E) HeLa cells were infected at MOI of 400 with Bhe ΔbepA-G expressing 3xFLAG-tagged BepCBhe or carrying the empty plasmid as a negative control for 24 h. Then cells were treated with inhibitors as specified below, followed by fixation and immunocytochemical staining. Specimen were then analyzed by fluorescence microscopy. F-actin is represented in white (scale bar = 50 μm). (B) Representative images of HeLa cells incubated for 2 h in the absence or presence of Rho inhibitor I at the indicated concentrations. (C) The mean fluorescence intensity of F-actin shown for conditions shown in (B) was quantified for each individual cell using CellProfiler. The graphs show the relative mean fluorescence intensity of the F-actin signal for the indicated condition normalized to the non-treated uninfected control. (D) Representative images of HeLa cells incubated for 1 h in the absence or presence of the ROCK inhibitor Y27632 at the indicated concentrations. (E) The mean fluorescence intensity of F-actin shown for conditions shown in (D) was quantified for each individual cell using CellProfiler. The graphs show the relative mean fluorescence intensity of the F-actin signal for the indicated condition normalized to the non-treated uninfected control. Data shown are representative results for three independent experiments. (PDF) [file ppat.1008548.s005.pdf]

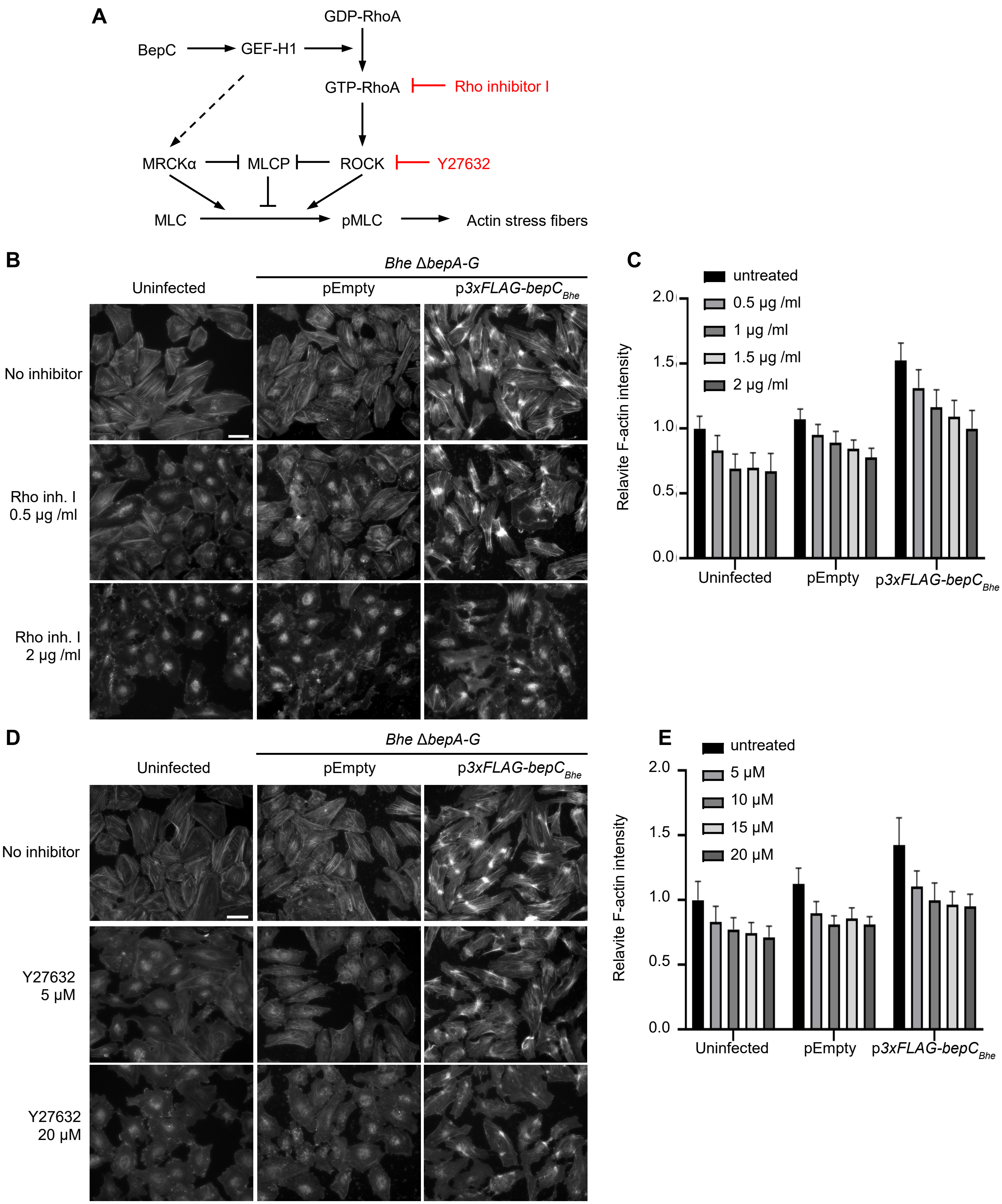
A
GDP-RhoA
BepC
GEF-H1
GTP-RhoA
Rho inhibitor I
MRCKα
MLCP
ROCK
Y27632
MLC
pMLC
Actin stress fibers
B
Bhe ΔbepA-G
Uninfected
pEmpty
p3xFLAG-bepC$_{Bhe}$
No inhibitor
Rho inh. I
0.5 µg /ml
Rho inh. I
2 µg /ml
C
untreated
0.5 µg /ml
1 µg /ml
1.5 µg /ml
2 µg /ml
Relavite F-actin intensity
2.0
1.5
1.0
0.5
0.0
Uninfected
pEmpty
p3xFLAG-bepC$_{Bhe}$
D
Bhe ΔbepA-G
Uninfected
pEmpty
p3xFLAG-bepC$_{Bhe}$
No inhibitor
Y27632
5 µM
Y27632
20 µM
E
untreated
5 µM
10 µM
15 µM
20 µM
Relavite F-actin intensity
2.0
1.5
1.0
0.5
0.0
Uninfected
pEmpty
p3xFLAG-bepC$_{Bhe}$

S5 Fig. Inhibition of RhoA/B/C or ROCK reduces actin stress fiber formation mediated by BepC$_{Bhe}$. (**A**) Proposed model of BepC-triggered actin stress fiber formation via the activation of the RhoA pathway and the targets of inhibitors used for validation. (**B-E**) HeLa cells were infected at MOI of 400 with *Bhe* Δ*bepA-G* expressing 3xFLAG-tagged BepC$_{Bhe}$ or carrying the empty plasmid as a negative control for 24 h. Then cells were treated with inhibitors as specified below, followed by fixation and immunocytochemical staining. Specimen were then analyzed by fluorescence microscopy. F-actin is represented in white (scale bar = 50 µm). (**B**) Representative images of HeLa cells incubated for 2 h in the absence or presence of Rho inhibitor I at the indicated concentrations. (**C**) The mean fluorescence intensity of F-actin shown for conditions shown in (B) was quantified for each individual cell using CellProfiler. The graphs show the relative mean fluorescence intensity of the F-actin signal for the indicated condition normalized to the non-treated uninfected control. (**D**) Representative images of HeLa cells incubated for 1 h in the absence or presence of the ROCK inhibitor Y27632 at the indicated concentrations. (**E**) The mean fluorescence intensity of F-actin shown for conditions shown in (D) was quantified for each individual cell using CellProfiler. The graphs show the relative mean fluorescence intensity of the F-actin signal for the indicated condition normalized to the non-treated uninfected control. Data shown are representative results for three independent experiments.
