# Supplementary material for: Bartonella effector protein C mediates actin stress fiber formation via recruitment of GEF-H1 to the plasma membrane
Source: PLoS Pathog. 2021 Jan 28;17(1):e1008548. doi: 10.1371/journal.ppat.1008548 (PMC7842960; doi:10.1371/journal.ppat.1008548)
Supplement: S6 Fig — (A) Proposed model of BepC-triggered actin stress fiber formation. (B) HeLa cells were infected with isogenic Bhe ΔbepA-G strains expressing FLAG-tagged BepCBhe wild-type or mutant variants, or carrying the empty plasmid at multiplicity of infection (MOI) of 200. After 48 h of infection, cells were fixed and immunocytochemically stained, followed by fluorescence microscopy analysis. Phosphorylated myosin light chain (pMLC) is represented in white (scale bar = 50 μm). BepCBhe**** = BepCBhe H146A, K150A, R154A, R157A. (C) The mean fluorescence intensity of F-actin shown for conditions shown in (B) was quantified for each individual cell using CellProfiler. Data are represented as dot plots with each data point corresponding to the average of all mean cell intensity values within one imaged site. Statistical significance was determined using Kruskal-Wallis test (**** corresponds to p-value ≤ 0.0001). (PDF) [file ppat.1008548.s006.pdf]

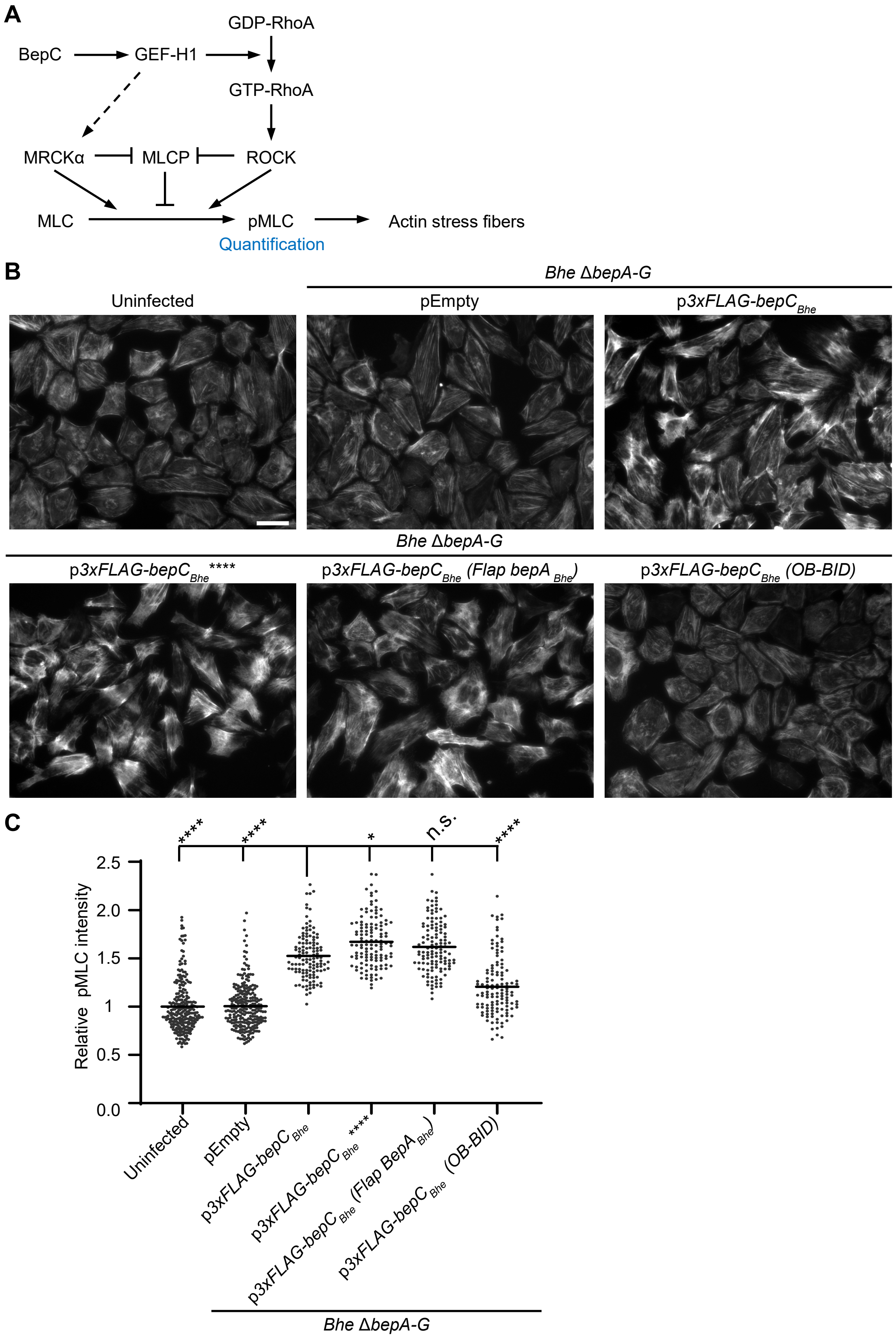
A
BepC
GEF-H1
GDP-RhoA
GTP-RhoA
MRCKα
MLCP
ROCK
MLC
pMLC
Actin stress fibers
Quantification
B
Bhe ΔbepA-G
Uninfected
pEmpty
p3xFLAG-bepC Bhe
Bhe ΔbepA-G
p3xFLAG-bepC Bhe ****
p3xFLAG-bepC Bhe (Flap bepA Bhe)
p3xFLAG-bepC Bhe (OB-BID)
C
****
****
*
n.s.
****
Relative pMLC intensity
2.5
2.0
1.5
1
0.5
0.0
Uninfected
pEmpty
p3xFLAG-bepC Bhe
p3xFLAG-bepC Bhe ****
p3xFLAG-bepC Bhe (Flap BepA Bhe)
p3xFLAG-bepC Bhe (OB-BID)
Bhe ΔbepA-G

S6 Fig. BepC$_{Bhe}$ induces a robust increase of myosin light chain phosphorylation. (**A**) Proposed model of BepC-triggered actin stress fiber formation. (**B**) HeLa cells were infected with isogenic *Bhe* Δ*bepA-G* strains expressing FLAG-tagged BepC$_{Bhe}$ wild-type or mutant variants, or carrying the empty plasmid at multiplicity of infection (MOI) of 200. After 48 h of infection, cells were fixed and immunocytochemically stained, followed by fluorescence microscopy analysis. Phosphorylated myosin light chain (pMLC) is represented in white (scale bar = 50 μm). BepC$_{Bhe}$**** = BepC$_{Bhe}$ H146A, K150A, R154A, R157A. (**C**) The mean fluorescence intensity of F-actin shown for conditions shown in (B) was quantified for each individual cell using CellProfiler. Data are represented as dot plots with each data point corresponding to the average of all mean cell intensity values within one imaged site. Statistical significance was determined using Kruskal-Wallis test (**** corresponds to p-value ≤ 0.0001).
