# Supplementary material for: Bartonella effector protein C mediates actin stress fiber formation via recruitment of GEF-H1 to the plasma membrane
Source: PLoS Pathog. 2021 Jan 28;17(1):e1008548. doi: 10.1371/journal.ppat.1008548 (PMC7842960; doi:10.1371/journal.ppat.1008548)
Supplement: S2 Table — (PDF) [file ppat.1008548.s008.pdf]

S2 Table. List of bacterial strains used in this study

Strain name	*Description*	Reference
MSE150	*Bhe ΔbepA-G*	Schulein et al., 2005
SIM B1-45	*Bhe ΔbepA-G / pFLAG-bepC$_{Bgr}$* (MSE150 containing pSIM051)	This work
SIM B1-46	*Bhe ΔbepA-G / pFLAG-bepC$_{Bqu}$* (MSE150 containing pSIM054)	This work
SIM B1-49	*Bhe ΔbepA-G / pFLAG-bepC$_{Bta}$* (MSE150 containing pSIM058)	This work
SIM B1-63	*Bhe ΔbepA-G / pFLAG-bepC$_{Btr}$* (MSE150 containing pSIM062)	This work
SIM B1-52	*Bhe ΔbepA-G / pEmpty* (MSE150 containing pBZ485_a_empty)	This work
SIM B2-06	*Bhe ΔbepA-G / pFLAG-bepC$_{Bhe}$* (MSE150 containing pSIM091)	This work
SIM B2-18	*Bhe ΔbepA-G / p3xFLAG-bepC$_{Bhe}$* (MSE150 containing pSIM107)	This work
SIM B2-38	*Bhe ΔbepA-G / p3xFLAG-bepC$_{Bhe}$***** (MSE150 containing pSIM127)	This work
SIM B2-42	*Bhe ΔbepA-G / p3xFLAG-bepC$_{Bhe}$ (OB-BID)* (MSE150 containing pSIM131)	This work
SIM B2-43	*Bhe ΔbepA-G / p3xFLAG-bepC$_{Bhe}$ (Flap bepA$_{Bhe}$)* (MSE150 containing pSIM132)	This work
LU B2-61	*Bhe ΔbepA-G Bhe ΔvirB4*	This work
JEN B1-38	*Bhe ΔbepA-G Bhe ΔvirB4* / p*Empty* (LUB261 containing pBZ485_a_empty)	This work
SIM B2-45	*Bhe ΔbepA-G Bhe ΔvirB4 /* p3x*FLAG-bepC$_{Bhe}$* (LU B2-61 containing pSIM091)	This work
JKE170	*E.coli* strain used to conjugate *Bartonella* expression vectors in *Bhe ΔbepA-G*	[1]

References:

Schulein R, Guye P, Rhomberg TA, Schmid MC, Schroder G, Vergunst AC, et al. A bipartite signal mediates the transfer of type IV secretion substrates of *Bartonella henselae* into human cells. Proc Natl Acad Sci U S A. 2005;102(3):856-61. Epub 2005/01/12. doi: 10.1073/pnas.0406796102. PubMed PMID: 15642951; PubMed Central PMCID: PMC545523.

Cunrath O, Meinel DM, Maturana P, Fanous J, Buyck JM, Saint Auguste P, et al. Quantitative contribution of efflux to multi-drug resistance of clinical *Escherichia coli* and *Pseudomonas aeruginosa* strains. EBioMedicine. 2019;41:479-87. Epub 2019/03/11. doi: 10.1016/j.ebiom.2019.02.061. PubMed PMID: 30852163; PubMed Central PMCID: PMCPMC6443642.
