# Supplementary material for: Bartonella effector protein C mediates actin stress fiber formation via recruitment of GEF-H1 to the plasma membrane
Source: PLoS Pathog. 2021 Jan 28;17(1):e1008548. doi: 10.1371/journal.ppat.1008548 (PMC7842960; doi:10.1371/journal.ppat.1008548)
Supplement: S3 Table — (PDF) [file ppat.1008548.s009.pdf]

S3 Table. List of *Bartonella* expression vectors used in this work

Internal name	Plasmid name	Description	Reference
pCD353		*Bartonella* spp. vector encoding GFP	Dehio et al., 1998[1]
pBZ485_a_empty	pEmpty	pSIM037 digested with NdeI, empty vector	This work
pSIM037	pBZ485_a_GFP	Derivative of pCD353 encoding GFP	This work
pSIM051	p*FLAG-bepC*$_{Bgr}$	pBZ485_a_empty encoding FLAG-BepC$_{Bgr}$	This work
pSIM054	p*FLAG-bepC*$_{Bqu}$	pBZ485_a_empty encoding FLAG-BepC$_{Bqu}$	This work
pSIM058	p*FLAG-bepC*$_{Bta}$	pBZ485_a_empty encoding FLAG-BepC$_{Bta}$	This work
pSIM062	p*FLAG-bepC*$_{Btr}$	pBZ485_a_empty encoding FLAG-BepC$_{Btr}$	This work
pSIM091	p*FLAG-bepC*$_{Bhe}$	pBZ485_a_empty encoding FLAG-BepC$_{Bhe}$	This work
pSIM107	p*3xFLAG-bepC*$_{Bhe}$	pBZ485_a_empty encoding 3xFLAG-BepC$_{Bhe}$	This work
pSIM127	p*3xFLAG-bepC*$_{Bhe}$****	pBZ485_a_empty encoding 3xFLAG-BepC$_{Bhe}$****	This work
pSIM131	p*3xFLAG-bepC*$_{Bhe}$ *(OB-BID)*	pBZ485_a_empty encoding 3xFLAG-BepC$_{Bhe}$ (OB-BID)	This work
pSIM132	p*3xFLAG-bepC*$_{Bhe}$ *(Flap BepA*$_{Bhe}$*)*	pBZ485_a_empty encoding 3xFLAG-BepC$_{Bhe}$ (Flap BepA$_{Bhe}$)	This work
pRS 25		Derivative of pRS14 used to generate a Δ*virB4* in frame deletion in *B. henselae*	Schmid et al., 2004

References:

Dehio M, Gomez-Duarte OG, Dehio C, Meyer TF. Vitronectin-dependent invasion ofepithelial cells by *Neisseria gonorrhoeae* involves alpha(v) integrin receptors. FEBS Lett. 1998;424(1-2):84-8. Epub 1998/04/16. doi: 10.1016/s0014-5793(98)00144-6. PubMed PMID: 9537520.

Schmid MC, Schulein R, Dehio M, Denecker G, Carena I, Dehio C. The VirB type VI secretion system of *Bartonella henselae* mediates invasion, proinflammatory activation and antiapoptotic protection of endothelial cells. Mol Microbiol. 2004; 52(1):81-92. Doi:10.1111/j.1365-2958.2003.03964.x. PubMed PMID: 15049812.
