# Supplementary material for: Bartonella effector protein C mediates actin stress fiber formation via recruitment of GEF-H1 to the plasma membrane
Source: PLoS Pathog. 2021 Jan 28;17(1):e1008548. doi: 10.1371/journal.ppat.1008548 (PMC7842960; doi:10.1371/journal.ppat.1008548)
Supplement: S4 Table — (PDF) [file ppat.1008548.s010.pdf]

**S4 Table.** List of eukaryotic expression plasmids used in this work

| Internal name | Plasmid name | Description | Reference |
|---|---|---|---|
| pFlag-CMV2 | pEmpty | eukaryotic expression vector encoding a short FLAG tag | Eastman Kodak, New Haven |
| pCMV5-eGFP-GEF-H1 | p*eGFP-GEF-H1* | eukaryotic expression vector encoding eGFP-GEF-H1 | Obtained from P. Nalbant; Kendel et al., 2002 |
| pX458 | pSpCas9(BB)-2A-GFP | eukaryotic expression vector encoding Cas9 and GFP | Ran et al., 2013 |
| pX459 | pSpCas9(BB)-2A-Puro | eukaryotic expression vector encoding Cas9, $Puro^R$ | Ran et al., 2013 |
| pSIM138 | | pX458 with sgRNA targeting Exon1 of GEF-H1 | This work |
| pSIM139 | | pX459 with sgRNA targeting Exon22 of GEF-H2 | This work |
| pSIM140 | | pX458 with sgRNA targeting Exon1 of MRCKa | This work |
| pSIM141 | | pX459 with sgRNA targeting Exon36 of MRCKa | This work |
| pSIM142 | p*FLAG-bepC*$_{Bgr}$ | pFlag-CMV2 encoding FLAG-BepC$_{Bgr}$ | This work |
| pSIM143 | p*FLAG-bepC*$_{Bqu}$ | pFlag-CMV2 encoding FLAG-BepC$_{Bqu}$ | This work |
| pSIM144 | p*FLAG-bepC*$_{Bta}$ | pFlag-CMV2 encoding FLAG-BepC$_{Bta}$ | This work |
| pSIM145 | p*FLAG-bepC*$_{Btr}$ | pFlag-CMV2 encoding FLAG-BepC$_{Btr}$ | This work |
| pSIM146 | p*FLAG-bepC*$_{Bhe}$ | pFlag-CMV2 encoding FLAG-BepC$_{Bhe}$ | This work |
| pSIM147 | p*3xFLAG-bepC*$_{Bhe}$ | pFlag-CMV2 encoding 3xFLAG-BepC$_{Bhe}$ | This work |
| pSIM148 | p*3xFLAG-bepC*$_{Bhe}$ *(FIC-OB)* | pFlag-CMV2 encoding 3xFLAG-BepC$_{Bhe}$ (FIC-OB) | This work |
| pSIM150 | p*3xFLAG-bepC*$_{Bhe}$**** | pFlag-CMV2 encoding 3xFLAG-BepC$_{Bhe}$ **** | This work |
| pSIM154 | p*3xFLAG-bepC*$_{Bhe}$ *(OB-BID)* | pFlag-CMV2 encoding 3xFLAG-BepC$_{Bhe}$ (OB-BID) | This work |
| pSIM160 | p*eGFP* | pCMV5 eukaryotic expression vector encoding eGFP | This work |

**References:**


Krendel M, Zenke FT, Bokoch GM. Nucleotide exchange factor GEF-H1 mediates cross-talk between microtubules and the actin cytoskeleton. Nat Cell Biol. 2002;4(4):294-301. doi: 10.1038/ncb773. PubMed PMID: 11912491.

Ran FA, Hsu PD, Wright J, Agarwala V, Scott DA, Zhang F. Genome engineering using the CRISPR-Cas9 system. Nat Protoc. 2013;8(11):2281-308. Epub 2013/10/26. doi: 10.1038/nprot.2013.143. PubMed PMID: 24157548; PubMed Central PMCID: PMCPMC3969860.
