# Supplementary material for: Bartonella effector protein C mediates actin stress fiber formation via recruitment of GEF-H1 to the plasma membrane
Source: PLoS Pathog. 2021 Jan 28;17(1):e1008548. doi: 10.1371/journal.ppat.1008548 (PMC7842960; doi:10.1371/journal.ppat.1008548)
Supplement: S5 Table — (PDF) [file ppat.1008548.s011.pdf]

S5 Table. Construction details for plasmids used in this work*

Plasmid	Insert template	PCR 1	PCR2	Backbone	Restriction
pSIM051	Genomic DNA *Bgr*	prSIM106/prSIM110	prSIM076/prSIM110	pSIM037	SacI/NotI
pSIM054	Genomic DNA *Bqu*	prSIM107/prSIM111	prSIM076/prSIM111	pSIM037	SacI/NotI
pSIM058	Genomic DNA *Bta*	prSIM155/prSIM156	prSIM076/prSIM156	pSIM037	SacI/NotI
pSIM062	Genomic DNA *Btr*	prSIM108/prSIM112	prSIM076/prSIM112	pSIM037	SacI/NotI
pSIM081	pSIM084	prSIM105/prSIM109	prSIM076/prSIM109	pSIM037	SacI/NotI
pSIM091	Genomic DNA *Bhe*	prSIM105/prSIM109	prSIM076/prSIM109	pSIM037	SacI/NotI
pSIM098	pSIM091	prSIM197/prSIM109	prSIM076/prSIM109	pSIM037	SacI/NotI
pSIM107	pSIM091	prSIM202/prSIM109	prSIM203/prSIM109	pSIM037	SacI/NotI
pSIM117	pSIM113	prSIM202/prSIM109	prSIM203/prSIM109	pSIM037	SacI/NotI
pSIM127	pSIM081	prSIM202/prSIM109	prSIM203/prSIM109	pSIM037	SacI/NotI
pSIM131	pSIM098	prSIM202/prSIM109	prSIM203/prSIM109	pSIM037	SacI/NotI
pSIM132	pSIM117	prSIM202/prSIM109	prSIM203/prSIM109	pSIM037	SacI/NotI
pSIM142	pSIM051	prSIM249/prSIM110	N.A.	pFlag-CMV2	SacI/NotI
pSIM143	pSIM054	prSIM249/prSIM111	N.A.	pFlag-CMV2	SacI/NotI
pSIM144	pSIM058	prSIM249/prSIM156	N.A.	pFlag-CMV2	SacI/NotI
pSIM145	pSIM062	prSIM249/prSIM112	N.A.	pFlag-CMV2	SacI/NotI
pSIM146	pSIM091	prSIM249/prSIM109	N.A.	pFlag-CMV2	SacI/NotI
pSIM147	pSIM107	prSIM248/prSIM109	N.A.	pFlag-CMV2	SacI/NotI
pSIM148	pSIM107	prSIM248/prSIM154	N.A.	pFlag-CMV2	SacI/NotI
pSIM150	pSIM127	prSIM248/prSIM109	N.A.	pFlag-CMV2	SacI/NotI
pSIM154	pSIM131	prSIM248/prSIM109	N.A.	pFlag-CMV2	SacI/NotI
pSIM160	pCMV5-eGFP-GEF-H1	prSIM259/prSIM260	N.A.	pCMV5-eGFP-GEF-H1	EcoRI/SacI

* Inserts were amplified from the indicated template with the indicated primers for PCR1 which, when indicated, served as a new template for PCR2. The inserts were ligated into the respective backbone via indicated restriction sites.
