# Supplementary material for: Bartonella effector protein C mediates actin stress fiber formation via recruitment of GEF-H1 to the plasma membrane
Source: PLoS Pathog. 2021 Jan 28;17(1):e1008548. doi: 10.1371/journal.ppat.1008548 (PMC7842960; doi:10.1371/journal.ppat.1008548)
Supplement: S6 Table — (PDF) [file ppat.1008548.s012.pdf]

S6 Table. Construction details for CRISPR/Cas expression constructs used in this study*

Plasmid	Annealed primers	Backbone	Restriction site
pSIM138	prSIM239/240	pX458	BbsI
pSIM139	prSIM241/242	pX459	BbsI
pSIM140	prSIM243/244	pX458	BbsI
pSIM141	prSIM245/246	pX459	BbsI

* Annealed primers were ligated into the indicated backbone using the BbsI restriction site
