# Supplementary material for: Bartonella effector protein C mediates actin stress fiber formation via recruitment of GEF-H1 to the plasma membrane
Source: PLoS Pathog. 2021 Jan 28;17(1):e1008548. doi: 10.1371/journal.ppat.1008548 (PMC7842960; doi:10.1371/journal.ppat.1008548)
Supplement: S7 Table — (PDF) [file ppat.1008548.s013.pdf]

S7 Table. List of primers used in this work

prSIM076	AGTCTAGAGCTCAAGAAGGAGATATACAAATGGACTACAAGGACGACGA
prSIM105	ATGGACTACAAGGACGACGATGACAAGATGTTAGAGCATAATTATCTGTAT
prSIM106	ATGGACTACAAGGACGACGATGACAAGATGTTAGAGCAAAATTATTTATATAA
prSIM107	ATGGACTACAAGGACGACGATGACAAGATGTTAGAGCATAATTATTTTTATAA
prSIM108	ATGGACTACAAGGACGACGATGACAAGATGTTAGAGCAAAATTATCTATATA
prSIM109	TGTCTAGCGGCCGCTTAGTTGGTAAGAGCCCTTGC
prSIM110	TGTCTAGCGGCCGCTTAGCCGGTAAGAGCAAGTG
prSIM111	TGTCTAGCGGCCGCTTAGCTGGTCAGAGCCATCG
prSIM112	TGTCTAGCGGCCGCTTAGCCTGTAAGAGCCAGTG
prSIM154	TGTCTAGCGGCCGCTCAAACATTGCTTTTTTCAAAGACTAT
prSIM155	ATGGACTACAAGGACGACGATGACAAGATGTTAGAGCAAAATTATCTGTAT
prSIM156	TGTCTAGCGGCCGCTTAGCCGGTAAGAGCTAGCG
prSIM197	ATGGACTACAAGGACGACGATGACAAGGAAATCAACGAACATATTGTTGT
prSIM202	ATGGACTACAAAGACCATGACGGTGATTATAAAGATCATGACATCGACTACAAGGACGACGATGA
prSIM203	AGTCTAGAGCTCAAGAAGGAGATATACAAATGGACTACAAAGACCATGAC
prSIM239	CACCGTCGAATCCCTCACGCGGGCG
prSIM240	AAACCGCCCGCGTGAGGGATTCGAC
prSIM241	CACCGGAGACGGAGAGCCGCGACG
prSIM242	AAACCGTCGCGGCTCTCCGTCTCC
prSIM243	CACCGACGGGCCCGCTCAGACCAA
prSIM244	AAACTTGGTCTGAGCGGGCCCGTC
prSIM245	CACCGAGCACTGACCGCGGGAGCT
prSIM246	AAACAGCTCCCGCGGTCAGTGCTC
prSIM248	AGTCTAGAGCTCGTTTAGTGAACCGTCAGAATTGATCTACCATGGACTACAAAGACCATGAC
prSIM249	AGTCTAGAGCTCGTTTAGTGAACCGTCAGAATTGATCTACCATGGACTACAAGGACGACGA
prSIM259	AGTCTAGAATTCATGGTGAGCAAGGG
prSIM260	AGTCTAGTCGACGGTACCCTTGTACAGCTCGT
