# Supplementary material for: Bartonella effector protein C mediates actin stress fiber formation via recruitment of GEF-H1 to the plasma membrane
Source: PLoS Pathog. 2021 Jan 28;17(1):e1008548. doi: 10.1371/journal.ppat.1008548 (PMC7842960; doi:10.1371/journal.ppat.1008548)
Supplement: S8 Table — (PDF) [file ppat.1008548.s014.pdf]

S8 Table. List of antibodies used in this work

**Antibody**	**Producer (reference)**	**Final dilution**
Rabbit anti-*Bartonella*	In-house (serum n° 2035)	IF: 1/1000
Rabbit anti-GEF-H1	ThermoFisher, PA5-32213	IF: 1/250, WB: 1/500
Rabbit anti-MRCKα	Abcam, ab96659	WB: 1/500
Rabbit anti-α tubulin	Cell signaling, # 2144	IF: 1/100
Mouse anti-Flag	Sigma, F1804	IF/WB: 1/1000
Mouse anti-pMLC	Cell signaling, # 3675	IF: 1/100
Goat anti-mouse AlexaFluor 488 (secondary)	Jackson Immuno, 115-545-146	IF: 1/250
Goat anti-mouse AlexaFluor 647 (secondary)	Invitrogen, A21240	IF: 1/250
Goat anti-rabbit Cy5 (secondary)	Jackson Immuno, 111-175-045	IF: 1/250
Goat anti-rabbit AlexaFluor 546 (secondary)	Invitrogen, A11035	IF: 1/250
Donkey anti-rabbit IgG, HRP linked (secondary)	GE Healthcare, NA934	WB: 1/1000
Sheep anti-mouse IgG, HRP linked (secondary)	GE Healthcare, NA931	WB: 1/1000
Rabbit anti- Na/K ATPase, HRP linked	Abcam, ab185065	WB: 1/1000
Rabbit anti-GFP	Molecular Probes, A11122	WB: 1/1000
